# Non-linear association of serum 25-hydroxyvitamin D with urinary albumin excretion rate in normoalbuminuric subjects

**DOI:** 10.1186/1471-2369-15-97

**Published:** 2014-06-24

**Authors:** Yun Jung Oh, Rae Woong Park, Dukyong Yoon, Myounghee Kim, Seung Seok Han, Hye Ryoun Jang, Hyosang Kim, Nam Ju Heo, Su-Kil Park, Hajeong Lee, Kwon Wook Joo, Chun-Soo Lim, Yon Su Kim, Dong Ki Kim

**Affiliations:** 1 Department of Internal Medicine, Seoul National University College of Medicine, 101 Daehak-ro, Jongro-gu Seoul 110-744, Korea; 2 Department of Internal Medicine, Cheju Halla General Hospital, Jeju, Korea; 3 Center for Clinical Epidemiology and Biostatistics, and Department of Biostatistics & Epidemiology and Center for Pharmacoepidemiololgy Research and Training, Perelman School of Medicine, University of Pennsylvania, Philadelphia, Pennsylvania, USA; 4 Department of Biomedical Informatics, Ajou University School of Medicine, Suwon, Korea; 5 Department of Dental Hygiene, College of Health Science, Eulji University, Seongnam, Korea; 6 Department of Internal Medicine, Samsung Medical Center, Sungkyunkwan University School of Medicine, Seoul, Korea; 7 Department of Internal Medicine, University of Ulsan college of Medicine, Asan Medical Center, Seoul, Korea; 8 Department of Internal Medicine, Healthcare System Gangnam Center, Seoul National University Hospital, Seoul, Korea

**Keywords:** Epidemiology, Low-grade albuminuria, Threshold, Vitamin D deficiency

## Abstract

**Background:**

Vitamin D deficiencies and increases in urinary albumin excretion (UAE) are both important and potentially related health problems; however, the nature of their relationship has not been established in normoalbuminuric subjects.

**Methods:**

We obtained data from 14,594 normoalbuminuric Korean adults who underwent voluntary health screenings. We used a generalized additive model to examine the threshold level for relationship between serum 25-hydroxyvitamin D [25(OH)D] and urinary-albumin creatinine ratio (UACR) levels. We conducted multivariate logistic regression for high-normal UAE (UACR, 10–29 mg/g), according to various categories of vitamin D status.

**Results:**

The generalized additive model confirmed a non-linear relationship between serum 25(OH)D and UACR levels, and the threshold concentration of 25(OH)D was 8.0 ng/mL after multivariate adjustment. Comparing subjects who fell into the lowest category of serum 25(OH)D levels with subjects who were in the reference range (the highest category), we observed that the multivariate adjusted odds ratio (OR) for high-normal UAE was significantly increased, regardless of the criteria used to categorize vitamin D levels: OR of the 1^st^ quartile over the 4^th^ quartile, 1.20 (95% CI, 1.04-1.39); OR of the 1.0-4.9^th^ percentile over the 50-100^th^ percentile, 1.56 (95% CI, 1.25-1.93); and OR of vitamin D deficiency group over vitamin D sufficiency group, 1.28 (95% CI, 1.08-1.52).

**Conclusions:**

We demonstrated that there was an inverse relationship between serum 25(OH)D less than 8.0 ng/mL and UACR in normoalbuminuric subjects, suggesting that severe vitamin D deficiency could cause an increase in UAE in subjects with normoalbuminuria.

## Background

Albuminuria is not only an independent prognostic marker for kidney disease, but it is also an established predictor of cardiovascular disease and all-cause mortality [1-3]. However, there has been considerable debate over the threshold level of urinary albumin excretion (UAE) that predicts adverse clinical outcomes. Recently, meta-analyses of general- and high-risk population cohorts have demonstrated that increased UAE, even within the normoalbuminuric range, especially a urinary albumin-creatinine ratio (UACR) of 10–29 mg/g, is an independent predictor of cardiovascular disease and all-cause mortality [4,5]. These noteworthy reports on the prognostic significance of the higher range of normoalbuminuria have challenged the previous definition of normoalbuminuria. Furthermore, given the high prevalence of the greater range of normoalbuminuria in the general population [6], we believe that exploring its risk factors might have important public health and clinical implications.

Vitamin D deficiency is an important public health issue because of its association with numerous adverse conditions [7]. The association of vitamin D with albuminuria has been suggested; however, most of these studies have been limited to subjects with chronic kidney disease or diabetes, which are susceptible to albuminuria [8-12]. Recently, general population studies have shown that vitamin D deficiency was associated with prevalent microalbuminuria and that vitamin D deficiency posed an increased risk of *de novo* microalbuminuria [13,14]. However, these studies did not consider high-normal UAE as a separate outcome variable, despite its obvious clinical significance and high prevalence. Moreover, previous studies did not consider a possible non-linear relationship between 25-hydroxyvitamin D [25(OH)D] and UACR levels, although recent large-scale studies have suggested a non-linear relationship between the risks of various clinical conditions and serum 25(OH)D levels [15-20].

Based on previous findings regarding the association between vitamin D and albuminuria, we postulated that low vitamin D levels might be a potential risk factor for high-normal UAE. In the present study, we examined the relationship between serum 25(OH)D and UACR levels in a large number of subjects who underwent voluntary health screenings. We aimed to estimate the threshold level of serum 25(OH)D less than which UAE would begin to increase.

## Methods

### Study population

We conducted this study using data from subjects who underwent voluntary health screenings at three university-affiliated hospitals, including Seoul National University Hospital (Seoul, Korea), Samsung Medical Center (Seoul, Korea), and Asan Medical Center (Seoul, Korea), between January 2008 and March 2012. Initially, we recruited a total of 16,870 subjects, who were aged ≥20 years old, and they underwent measurements of serum 25(OH)D and UACR. From that group, we excluded 2,276 subjects with outlying values of serum 25(OH)D (>125 ng/mL, N = 3) or UACR ≥30 mg/g (N = 2,276). A total of 14,594 participants were included in the study analysis. This study was approved by the institutional review boards of the three hospitals and performed in accordance with the principle of Helsinki Declaration. The IRBs approved waiver of the requirement for obtaining written informed consent because this study was conducted by retrospective chart review and presented no more than minimal risk to participants.

### Normalization of laboratory tests

The quantile normalization method [21,22] was applied to combine the laboratory test results of the three participating institutions, using a Java programming tool (Eclipse, version 3.7.1, IBM, Riverton, NJ). The quantile normalization method is a technique that redistributes values of datasets to have same statistical properties. The quantile normalization makes datasets have identical distribution by averaging values at the same ranking in each dataset. Each datum of a distribution was sorted in ascending order, and the data in the same rank were averaged together. The averaged value was set as the new normalized value. To determine the reference range, all of the laboratory test results were subdivided by reference range, and subsequently, each stratum was independently normalized.

### Vitamin D and albuminuria

We measured serum 25(OH)D levels at a single baseline visit. Serum 25(OH)D levels were measured using a radioimmunoassay kit (BIOSOURCE, Brussels, Belgium) and a gamma-counter (COBRA 5010 Series Quantum, PACKARD, Meriden, CT) at two medical centers (Seoul National University Hospital and Asan Medical Center), while at Samsung Medical Center, these levels were measured by liquid chromatography-tandem mass spectrometry assay, using a mass spectrometer (6460 Triple Quadrupole mass spectrometer, Agilent, Santa Clara, CA). We categorized serum 25(OH)D levels by quartiles. We also divided the subjects into four groups by percentile categories of 25(OH)D concentrations (1.0-4.9^th^; 5.0-24.9^th^, 25.0-49.9^th^ and 50.0-100.0^th^ percentile). Additionally, we assigned categories of serum 25(OH)D levels according to generally used clinical cut-off points (vitamin D deficiency, <15.0 ng/mL; insufficiency, 15.0-29.9 ng/mL; sufficiency, >30 ng/mL) [23,24]. For an assessment for UACR, urine albumin and creatinine were measured from a single random voided urine sample. Urine albumin levels were measured by immunoturbimetric assay (ALBT2, Roche, Basel, Switzerland). Urine creatinine levels were measured using the Jaffe method (CREJ2, Roche). We classified the status of UAE into two groups according to UACR levels. Normal UAE was defined as UACR <10 mg/g and high-normal UAE was defined as UACR 10–29 mg/g.

### Study variables

Demographic and clinical data included age, sex, body mass index (BMI), blood pressure, blood and urine chemistry analysis, and medication history, including vitamin D supplements and renin angiotensin system (RAS) blockers. We classified time points into four seasons as follows; spring (March to May); summer (June to August); autumn (September to November); and winter (December to February). Additionally, we assembled information about medical co-morbidities. Hypertension was defined as blood pressure ≥140/90 mmHg; a reported history of hypertension or those individuals who received antihypertensive medications were documented. Diabetes was defined as a reported history of diabetes, including individuals who received oral hypoglycemic agents or insulin. The estimated glomerular filtration rate (eGFR) was calculated using four-variable Modification of Diet in Renal Disease equation [25].

### Statistical analysis

All of the analyses and calculations were performed using SPSS Statistics software (version 19.0, Chicago, IL) and R software, version 2.15.1, with the mgcv package (The Comprehensive R Archive Network: http://cran.r-project.org). Continuous variables with normal distributions are presented as the means ± SDs and were compared using Student’s *t*-test. Non-normally distributed continuous variables are expressed as the medians (25-75% interquartile ranges) and were compared using the Mann–Whitney *U*-test. Categorical variables are expressed as frequencies or percentages, and comparison between groups was performed using the chi-squared test for dichotomized variables. We conducted logistic regression analysis to identify the independent effects of serum 25(OH)D levels on the risk of high-normal UAE. Two-sided *P* values were reported, and statistical significance was defined as *P* < 0.05.

A Gaussian model was adapted to evaluate the effects of serum 25(OH)D levels on UACR levels. To investigate the continuous relationship between serum 25(OH)D and UACR levels, we used generalized additive models (GAMs) with a smoothing function for UACR levels [26]. In addition to univariate analysis, we performed multivariate analysis, constructing multiple models adjusted for relevant covariates in a stepwise manner. To estimate the threshold of the serum 25(OH)D concentration for each model, we first selected the serum 25(OH)D range and fitted the GAM to quantify the relationship between serum 25(OH)D and UACR levels. Akaike’s information criterion (AIC) was used to determine a primary measurement of model fit; a lower value of AIC indicated a better model fit. The threshold of serum 25(OH)D was chosen, based on the best fit determined by AIC among the models, with threshold values that differed in 0.2-increments. We plotted the optimal criterion on the GAM model. Predicted probability was calculated from the multivariate logistic regression model. We presented curves of predicted probability and 95% CIs using the lowess regression.

## Results

### Clinical characteristics

**Figure 1:**
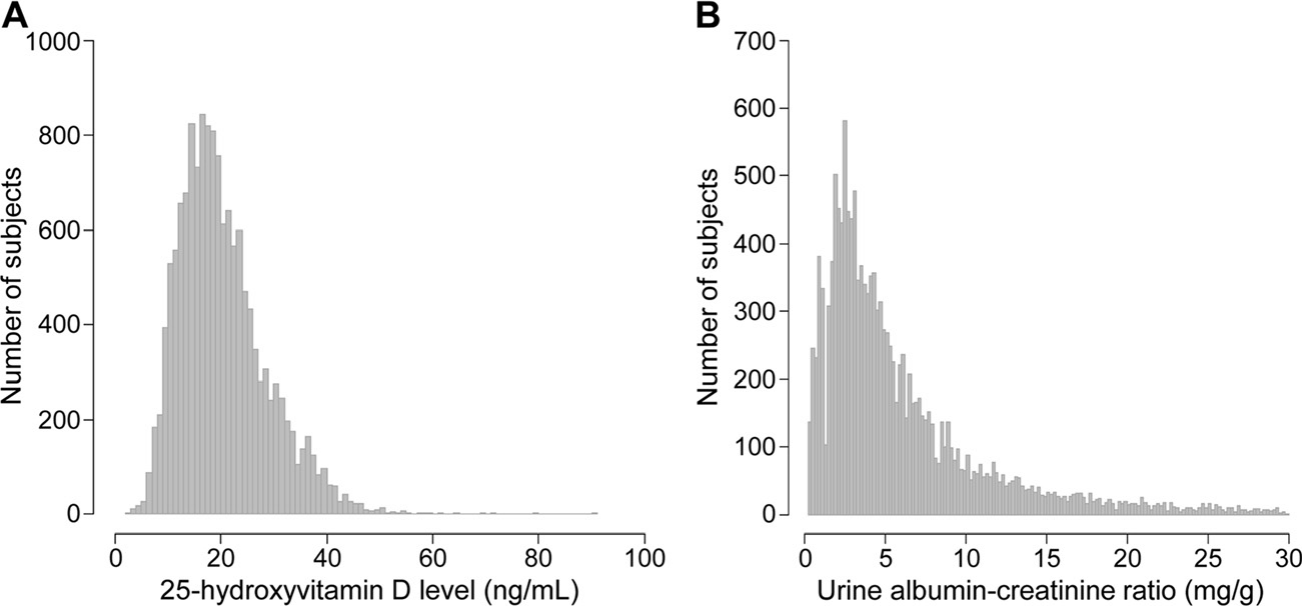
Distribution of serum 25-hydroxyvitamin D levels (A) and urinary albumin-creatinine ratios (B).

A total of 14,594 subjects were included in this study. The mean age was 54 years old, and 64.7% of participants were male. The seasonal distribution of the examination was nearly even across the entire examination period. The serum 25(OH)D levels were normally distributed (Figure 1A). However, UACR showed a highly skewed distribution (Figure 1B). Table 1 presents the clinical characteristics of study population. The mean serum 25(OH)D and eGFR levels were 20.4 ± 8.4 ng/mL and 85.8 mL/min/1.73 m^2^, respectively, and the median value of UACR was 4.3 mg/g. Overall, 28.0% of participants had vitamin D deficiency. The serum 25(OH)D level was lower in high-normal UAE than in normal UAE. The hemoglobin level was also lower in high-normal UAE compared to normal UAE; however, the serum albumin and eGFR levels were not significantly different between the groups. Additionally, the prevalence of diabetes and hypertension in subjects with high-normal UAE was significantly higher compared to those with normal UAE.

**Table 1:** Clinical characteristics of study population

	**Total**	**UACR <10 mg/g**	**UACR 10–29 mg/g**	**P value**	**Serum 25(OH)D ≥15 mg/**	**Serum 25(OH)D <15 mg/g**	**P value**
	(N = 14,594)	(N = 12,170)	(N = 2,424)		(N = 10,505)	(N = 4,089)	
Age (years)	54.0 ± 9.0	53.4 ± 8.5	56.6 ± 9.8	<0.01	54.3 ± 8.7	53.0 ± 9.0	<0.01
Male (N/%)	9,441/64.7	8,168/67.1	1,276/52.5	<0.01	7,237/68.9	2,204/53.9	<0.01
Season (N/%)				<0.01			<0.01
Spring	3,813/26.1	3,195/26.3	619/25.5		2,550/24.3	1,263/30.9	
Summer	3,772/25.8	3,217/26.4	557/22.9		3,004/28.6	768/18.8	
Autumn	3,620/24.8	3,010/24.7	611/25.2		2,853/27.2	767/18.8	
Winter	3,389/23.2	2,748/22.6	642/26.4		2,098/20.0	1,291/31.6	
BMI	24.39 ± 2.97	24.36 ± 2.90	24.53 ± 3.25	0.15	24.54 ± 2.90	24.01 ± 3.12	<0.01
25(OH)D (ng/mL)	20.4 ± 8.4	20.5 ± 8.4	20.0 ± 8.7	<0.01	23.8 ± 7.4	11.7 ± 2.3	<0.01
UACR (mg/g)	4.3 (2.5-7.7)	3.6 (2.2-5.6)	14.5 (11.9-19.1)	<0.01	4.2 (2.4-7.5)	4.6 (2.7-8.2)	<0.01
Hemoglobin (g/dL)	14.5 ± 1.5	14.5 ± 1.5	14.2 ± 1.7	<0.01	14.6 ± 1.5	14.2 ± 1.6	<0.01
Serum albumin (g/dL)	4.4 ± 0.3	4.4 ± 0.3	4.4 ± 0.3	0.89	4.4 ± 0.3	4.4 ± 0.3	<0.01
Serum calcium (mg/dL)	9.1 ± 0.4	9.0 ± 0.4	9.1 ± 0.4	<0.01	9.1 ± 0.4	9.0 ± 0.4	<0.01
Serum phosphorus (mg/dL)	3.5 ± 0.5	3.4 ± 0.5	3.5 ± 0.6	<0.01	3.4 ± 0.5	3.5 ± 0.5	<0.01
hs-CRP (mg/dL)	0.10 (0.00-0.10)	0.10 (0.00-0.10)	0.10 (0.00-0.20)	<0.01	0.10 (0.00-0.10)	0.10 (0.00-0.10)	0.003
Total cholesterol	189 ± 35	189 ± 34	188 ± 39	0.38	190 ± 35	187 ± 36	<0.01
LDL-cholesterol	121 ± 33	122 ± 32	120 ± 35	0.153	122 ± 33	120 ± 33	<0.01
HDL-cholesterol	54 ± 13	54 ± 13	54 ± 14	0.762	54 ± 13	54 ± 13	0.768
Triglyceride	120 ± 74	119 ± 72	126 ± 82	<0.01	122 ± 74	116 ± 74	<0.01
eGFR (mL/min/1.73 m^2^)	85.8 ± 18.6	86.3 ± 17.3	86.7 ± 20.7	0.365	85.1 ± 17.4	89.4 ± 18.7	<0.01
Diabetes	1,348/9.2	1,001/8.2	348/14.3	<0.01	972/9.3	376/9.2	0.914
Hypertension	1,721/11.8	1,289/10.6	435/17.9	<0.01	1,279/12.2	442/10.8	0.022
RAS blocker	1,013/6.9	765/6.3	250/10.3	<0.01	755/7.2	258/6.3	0.061
Vitamin D supplementation	1,012/6.9	766/6.8	247/11.0	<0.01	700/7.2	312/8.3	0.038

*Abbreviations*: *25(OH)D* 25-hydroxyvitamin D, *BMI* body mass index, *UACR* urine albumin-creatinine ratio, *hs-CRP* high sensitivity-C-reactive protein, *LDL* low density lipoprotein, *HDL* how density lipoprotein, *eGFR* estimated glomerular filtration rate, *RAS* renin angiotensin system. Variables are expressed as the means ± SD or median (25^th^,75^th^ percentiles).

### Associations of serum 25(OH)D levels with UACR and threshold 25(OH)D values for their associations with albuminuria

**Figure 2:**
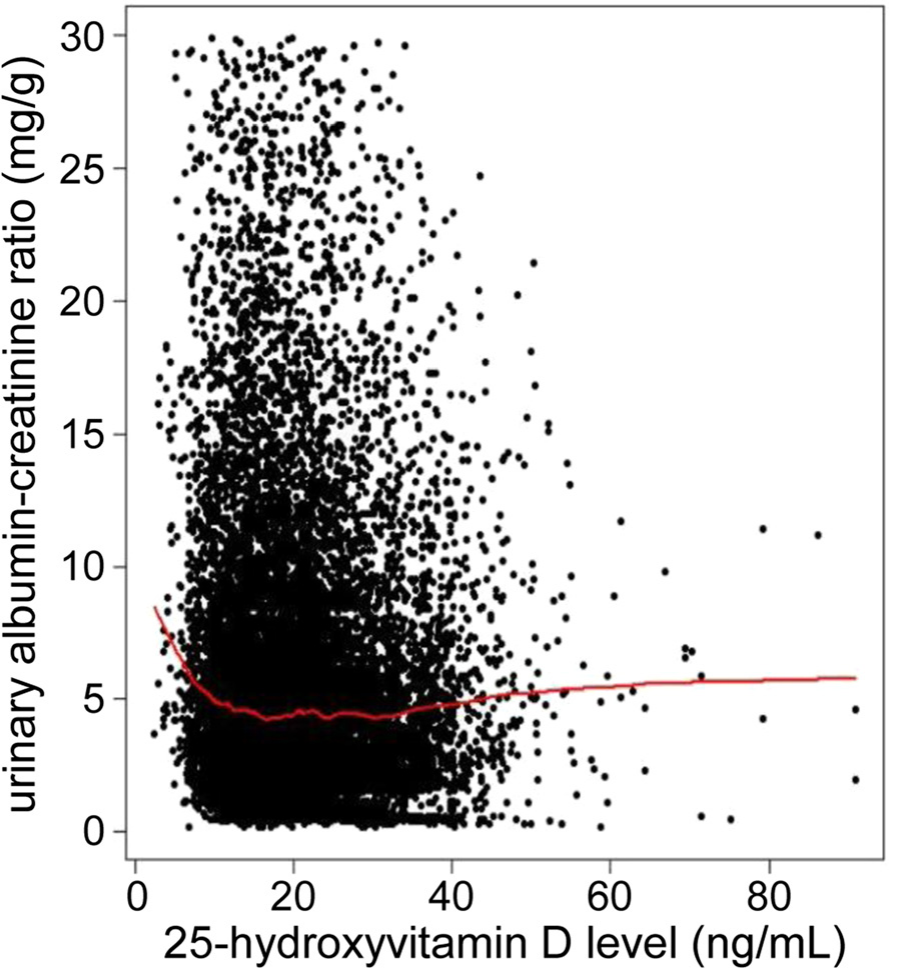
**Scatter plot with the lowess regression curve between serum 25-hydroxyvitamin D and urinary albumin-creatinine ratio.** A single unbroken line represents the mean value of the urinary albumin-creatinine ratio.

**Figure 3:**
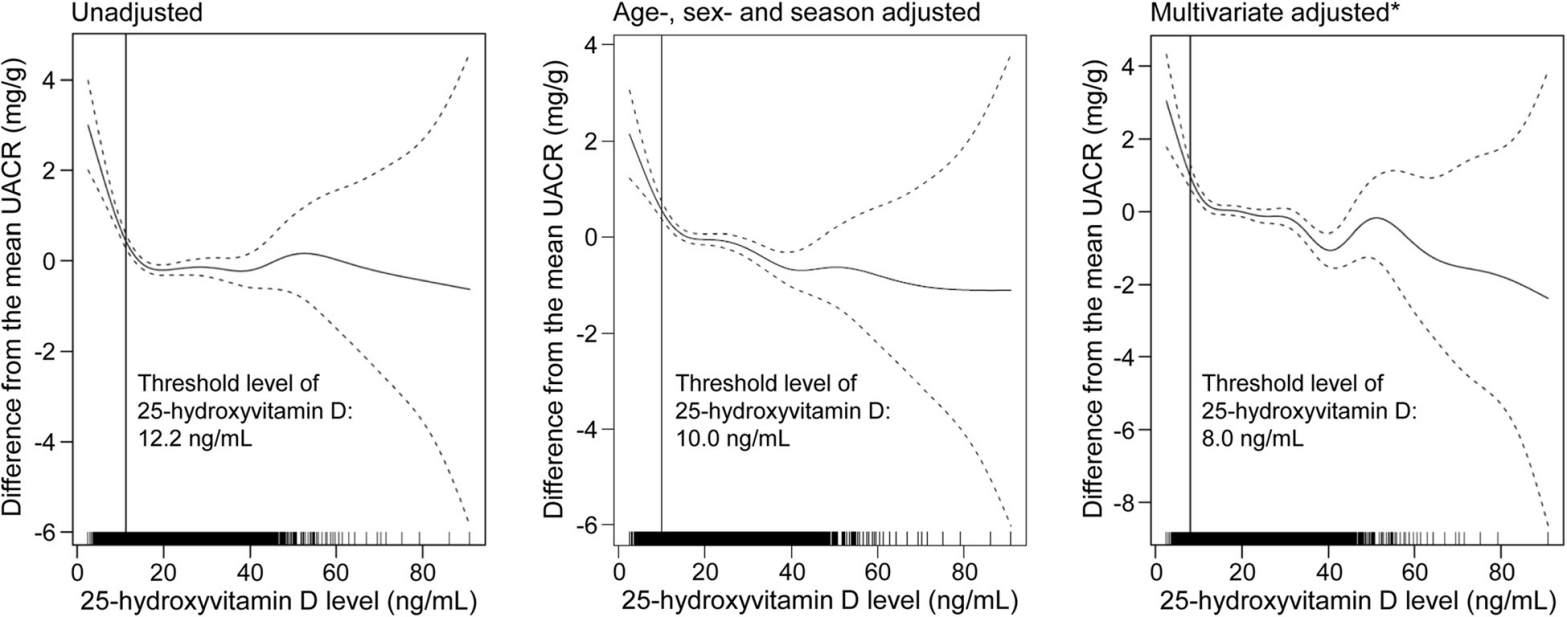
**25-hydroxyvitamin D threshold values suggested by generalized additive model plots.** The dashed line indicates 95% confidential intervals for value of the smoothed urine albumin creatinine ratio *Adjusted for age, sex, season, body mass index, hemoglobin, estimated glomerular filtration rate, hypertension, diabetes, use of vitamin D supplements, and/or renin-angiotensin system blocker, serum albumin, calcium, phosphorus, high-density lipoprotein-cholesterol, triglyceride, and high sensitivity-C-reactive protein. UACR, urinary albumin-creatinine ratio.

We explored the continuous relationship between serum 25(OH)D and UACR levels. As shown in the lowess line of Figure 2, there was no specific association of serum 25(OH)D with UACR levels until 25(OH)D levels decreased to approximately 15 ng/mL; however, the UACR level increased with decreasing serum 25(OH)D to approximately less than 15 ng/mL of serum 25(OH)D level. Based on the findings, we investigated the non-linear relationship between serum 25(OH)D and UACR levels, and we estimated the threshold level of serum 25(OH)D less than which UACR levels started to increase. To estimate the threshold, we used GAM, and we made adjustments for relevant covariates, including age, sex, season, BMI, hemoglobin, eGFR, hypertension, diabetes, use of vitamin D supplements and RAS blockers, serum albumin, calcium, phosphorus, high-density lipoprotein (HDL) cholesterol, triglycerides, and high-sensitivity-C-reactive protein (hs-CRP), in a stepwise manner. In each model, we used the AIC to assess model fit, and we finally selected the best-adapted model for estimating the threshold. Figure 3 shows the unadjusted, age-, sex-, and season-adjusted, and multivariate-adjusted GAM plots used to identify the threshold level of 25(OH)D that predicted changes in UACR levels. In the best adapted model, adjusted for potential confounding factors, 8.0 ng/mL of serum 25(OH)D had the lowest AIC value: the difference from the mean UACR began to increase as the serum 25(OH)D level decreased to less than the threshold level of 8.0 ng/mL.

### Risk of high-normal UAE according to the serum 25(OH)D levels

**Figure 4:**
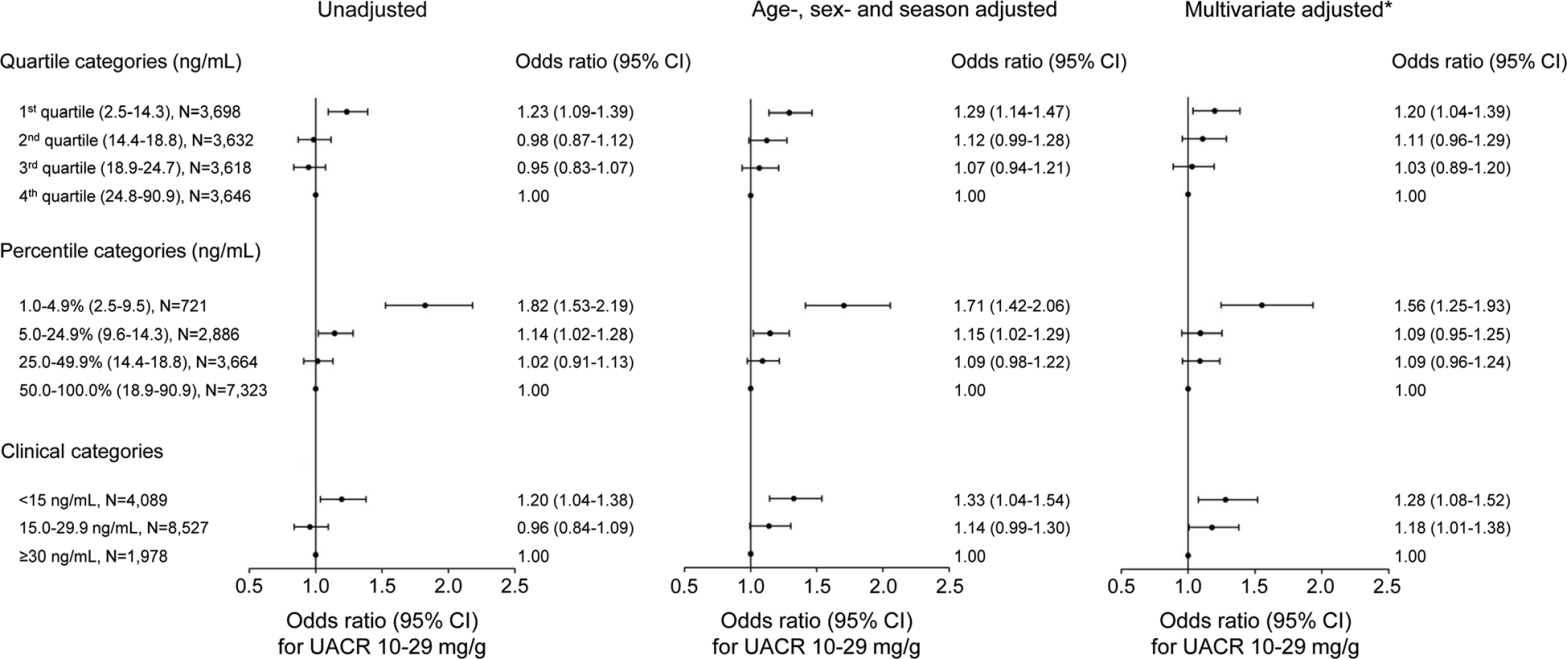
**Odds ratios for high-normal urinary albumin excretion (UACR 10–29 mg/g) as a function of serum 25-hydroxyvitamin D levels by quartile categories (top), percentile categories (middle), and clinical categories (bottom).** Black round dots represent odds ratios, and error bars represent the 95% CIs. *Adjusted for age, sex, season, body mass index, hemoglobin, estimated glomerular filtration rate, hypertension, diabetes, use of vitamin D supplements, and/or renin-angiotensin system blocker, serum albumin, calcium, phosphorus, high-density lipoprotein-cholesterol, triglyceride, and high sensitivity-C-reactive protein. UACR, urinary albumin-creatinine ratio.

**Figure 5:**
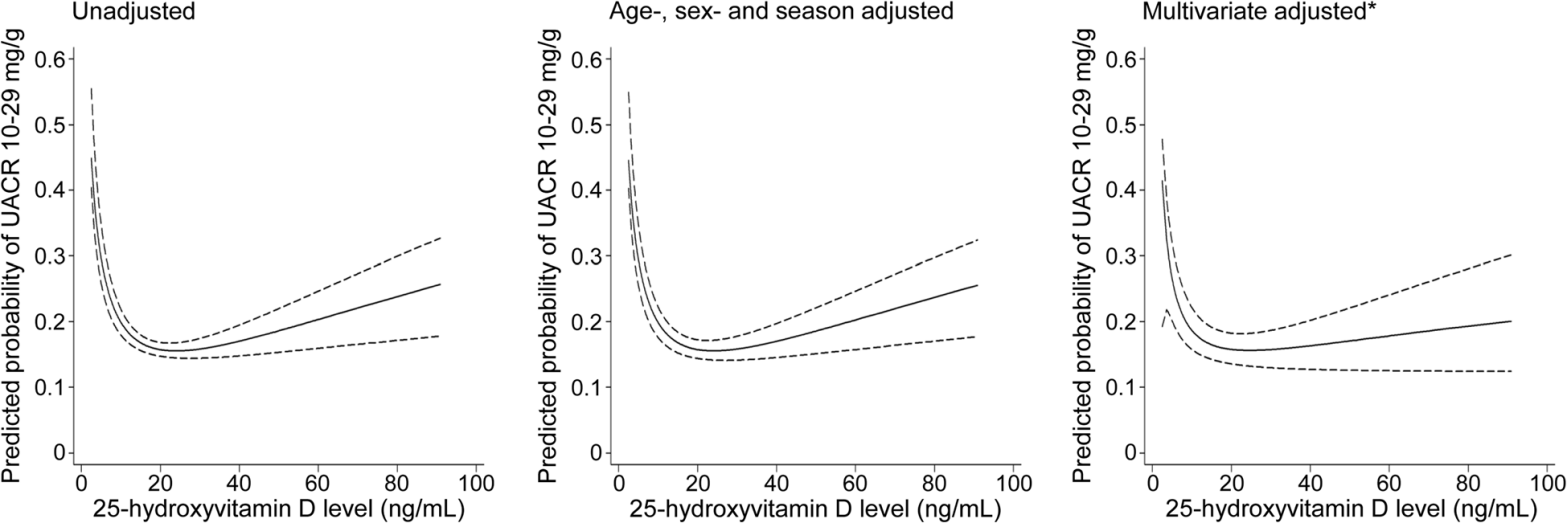
**Predicted prevalence curve for high-normal urinary albumin excretion in subjects with UACR < 30 mg/g (n = 14,594) according to serum 25-hydroxyvitamin D levels. Dashed lines represent 95% CIs.** *Adjusted for age, sex, season, body mass index, hemoglobin, estimated glomerular filtration rate, hypertension, diabetes, use of vitamin D supplements, and/or renin-angiotensin system blocker, serum albumin, calcium, phosphorus, high-density lipoprotein-cholesterol, triglyceride, and high sensitivity-C-reactive protein. UACR, urinary albumin-creatinine ratio.

We investigated whether serum 25(OH)D levels were significantly associated with the prevalence of high-normal UAE, using a logistic regression model. A stepwise increase in the prevalence of high-normal UAE was observed with decreasing serum 25(OH)D levels, which were indicated by quartiles, percentiles, and clinical categories (Figure 4). However, we observed a significant increase in high-normal UAE in only the lowest category of serum 25(OH)D, which indicated very low vitamin D levels. Adjusting for multiple covariates, including age, sex, season, BMI, hemoglobin, eGFR, hypertension, diabetes, use of vitamin D supplements and RAS blockers, serum albumin, calcium, phosphorus, HDL cholesterol, triglycerides, and hs-CRP, all of the lowest categories of serum 25(OH)D levels, by quartile, percentile, and clinical category, were significantly associated with the risk of high-normal UAE. The OR of the lowest quartile over the highest quartile was 1.20 (95% CI, 1.04-1.39), the OR of the 1.0-4.9% over the 50.0-100.0% was 1.56 (95% CI, 1.25-1.93), and the OR of subjects with vitamin D deficiency over those with vitamin D sufficiency was 1.28 (95% CI, 1.08-1.52). Stratified analyses according to the presence or absence of diabetes showed similar patterns. In the subjects with diabetes, multivariate-adjusted odds of high-normal UAE were significantly higher in the lowest serum 25(OH)D quartile and in vitamin D deficiency defined by the clinical category. The risk of high-normal UAE in non-diabetic subjects with vitamin D deficiency was also significantly higher than that in non-diabetic subjects with vitamin D sufficiency (Additional file 1: Figure S1).Figure 5 presents the predicted probability of high-normal UAE based on serum 25(OH)D levels. After full adjustments for age, sex, season, BMI, hemoglobin, eGFR, hypertension, diabetes, use of vitamin D supplements and RAS blockers, serum albumin, calcium, phosphorus, HDL cholesterol, triglycerides, and hs-CRP, the predicted probability of high-normal UAE increased non-linearly with decreasing serum 25(OH)D levels, particularly at approximately the threshold levels of serum 25(OH)D.

## Discussion

In the present study, conducted in a large number of health-screening subjects, we demonstrated a non-linear relationship between serum 25(OH)D and UACR levels in normoalbuminuric subjects, and we identified the threshold level of serum 25(OH)D that predicted an increase in UAE, independent of confounding variables. Because the serum 25(OH)D threshold was lower than the generally accepted lower limit of the normal range, the present study indicates that severe vitamin D deficiency could cause an increase in UAE. Additionally, we demonstrated that the risk of high-normal UAE (UACR 10–29 mg/g) increased as the serum 25(OH)D level decreased; however, the trend was not linear. Thus, the present study raises the important issue that vitamin D could be a potential therapeutic option for the management or prevention of increases in UAE in normoalbuminuric subjects with severe vitamin D deficiency.

Increases in UAE, even within the normoalbuminuric range, constitute a well-known risk factor for adverse cardiovascular outcomes in high-risk individuals [27,28]. More recently, high-normal UAE has become a critical public health issue because high-normal UAE has been associated with the risk of cardiovascular disease and all-cause mortality in the general population [4,5,29]. Additionally, because large-scale, population-based studies have documented a high prevalence of high-normal UAE, ranging from 13% to 23% [30-33], its public health aspects require greater attention. However, despite its significance for public health, there have been few studies investigating its causative or potentially modifiable risk factors, other than traditional ones, such as diabetes, hypertension, or obesity. Given that low-grade albuminuria reflects generalized vascular dysfunction via impaired endothelial function, activation of the RAS, and chronic low-grade inflammation [34-37]. conditions relevant to the development of these pathogenic processes could constitute potential risk factors for high-normal UAE. In this respect, vitamin D deficiency is a likely mechanism for the increase in UAE, as it has been shown to play a crucial role in vascular dysfunction. Recent studies have suggested that flow-mediated dilatation, which is a standard tool for measuring endothelial function, was significantly impaired in subjects with vitamin D deficiency [38,39]. Vitamin D deficiency also adversely affected vascular function by inappropriate activation of the RAS [40], and chronic inflammation [41]. These processes might, in turn, increase UAE, even in the absence of evident kidney disease. The data presented show that severe vitamin D deficiency was significantly, but non-linearly, correlated with high-normal UAE, even after adjustment for traditional risk factors for albuminuria. Similar to our results, a previous study found that low serum 25(OH)D levels were associated with an increased prevalence of albuminuria in a general population [14]. However, in that study, microalbuminuria (defined as UACR of 17–249 mg/g for men and 25–354 mg/g for women), but not high-normal UAE, was considered to be a dependent variable, so the reference group included a large number of subjects with high-normal UAE, which is a proven risk factor for adverse outcomes.

Recently, mounting evidence has suggested that the inverse associations between serum 25(OH)D levels and risk factors in cardiovascular outcomes might be non-linear, involving a threshold effect [15,18,19]. Given the close pathophysiological association between albuminuria and cardiovascular disease, serum 25(OH)D levels could also have a threshold effect on levels of UAE. The present results of GAM, which is a non-parametric regression method used to address non-linearity between variables [26], are consistent with the hypothesis that the association between serum 25(OH)D and UACR levels is non-linear. However, the threshold serum 25(OH)D concentration in this study (8 ng/mL) was much lower than that reported in previous studies that explored the associations between serum 25(OH)D levels and risk factors for cardiovascular disease. Previous studies have demonstrated that the risk for cardiovascular disease began to increase significantly at less than 15 to 30 ng/mL of serum 25(OH)D [15,18,19], which is relevant to the current definition of vitamin D deficiency. Considering the high prevalence of vitamin D deficiency in the general population, a substantial number of subjects would be expected to be at vitamin D deficiency-related risk for cardiovascular disease. However, most randomized, controlled trials with vitamin D supplementation have failed to demonstrate improved vascular function or cardiovascular outcomes [42-44], suggesting that low vitamin D levels simply reflect health status, rather than directly causing disease. An alternate explanation for the discrepancy is that vitamin D plays an independent role in the development of cardiovascular disease or vascular dysfunction, when its levels decrease to less than very low levels. This hypothesis was supported by a recent large-scale, population-based study, which demonstrated that only a very low vitamin D level (<5^th^ percentile of serum 25(OH)D) significantly increased the risk of fatal ischemic heart disease after adjustment for traditional cardiovascular risk factors [39]. In accordance with the previous study, the present study also demonstrated that the 25(OH)D threshold levels decreased after adjustment for the traditional cardiovascular risk factors in GAM analysis. Additionally, only the lowest category of serum 25(OH)D was correlated with elevated odds of high-normal UAE after multivariate adjustment. These findings suggest that vitamin D levels related to the pathogenesis of low-grade albuminuria might be lower than the lower limit of the normal range of vitamin D. This outcome could be one of the possible indirect explanations for the negative results of randomized, controlled trials regarding the effects of vitamin D supplementation on vascular function, as low-grade albuminuria could reflect generalized vascular dysfunction.

There were several limitations to the present study. First, UAE was measured from only a single random urine sample. Individual variability in UACR levels could have caused measurement error for UAE. Second, there was a lack of information on vitamin D dietary intake and outdoor physical activities, which exert effects on vitamin D status. Third, the current investigation was cross-sectionally designed. We could not establish a causative or temporal relationship between vitamin D levels and UAE. Thus, we could not demonstrate whether use of vitamin D could reduce urinary albumin loss in a healthy population with very low degrees of UAE. Finally, the laboratory test methods were not consistent among the institutions. Thus, to combine laboratory test results from different institutions, a normalization process, which transformed different distributions into identical distributions in statistical properties, was indispensable. We used quantile normalization, which is the most widely used normalization method for genetic studies [22]. This method makes the distribution of different datasets as similar as possible while conserving the meaning of original values. To consider the reference range of laboratory tests, we partitioned each datasets into three groups (upper range, within range and below range), and each of them were independently normalized. By doing this, we could normalize the datasets while conserving the clinical meaning and considering the reference range. Because this method does not assume any parametric distributions, the method can be applied to any laboratory test results with various distributions.

## Conclusions

Severe vitamin D deficiency was independently associated with an increased prevalence of high-normal UAE, which has tended to be overlooked. Based on the clinical significance of low-grade albuminuria as a predictor of vascular dysfunction and cardiovascular mortality [4,29], the clinical implications of severe vitamin D deficiency in public health as a modifiable risk factor for low-degree albuminuria might be substantial. We confirmed a non-linear relationship between vitamin D levels and UAE, as well as the threshold (8 ng/mL) less than which vitamin D levels began to be associated with an increase in UAE. Additional clinical studies are warranted to validate our study and to evaluate whether vitamin D therapy could reduce urinary albumin loss in normoalbuminuric individuals.

## Competing interests

The authors declare that they have no competing interests.

## Authors’ contributions

YJO, RWP, and DKK conceived of the design of this research, and helped to draft the manuscript. DKK supervised this project. YJO, HRJ, HK, HL, NJH, and SKP collected the data. YJO, DY, MK, and SSH analyzed the data. RWP and KWJ interpreted the results. YSK and CSL gave conceptual advice and comments on the manuscript. All authors read and approved the final manuscript.

## Pre-publication history

The pre-publication history for this paper can be accessed here:

http://www.biomedcentral.com/1471-2369/15/97/prepub

## Supplementary Material

Additional file 1: Figure S1 Odds ratios for high-normal urinary albumin excretion (UACR 10–29 mg/g) as a function of serum 25-hydroxyvitamin D levels by quartile categories (top), percentile categories (middle), and clinical categories (bottom) in subjects without diabetes and with diabetes. Dashed lines represent 95% CIs. *Adjusted for age, sex, season, body mass index, hemoglobin, estimated glomerular filtration rate, hypertension, diabetes, use of vitamin D supplements, and/or renin-angiotensin system blocker, serum albumin, calcium, phosphorus, high-density lipoprotein-cholesterol, triglyceride, and high sensitivity-C-reactive protein. UACR, urinary albumin-creatinine ratio. Click here for file
