# Supplementary figures and images for: Non-linear association of serum 25-hydroxyvitamin D with urinary albumin excretion rate in normoalbuminuric subjects
Source: BMC Nephrol. 2014 Jun 24;15:97. doi: 10.1186/1471-2369-15-97 (PMC4079922; doi:10.1186/1471-2369-15-97)

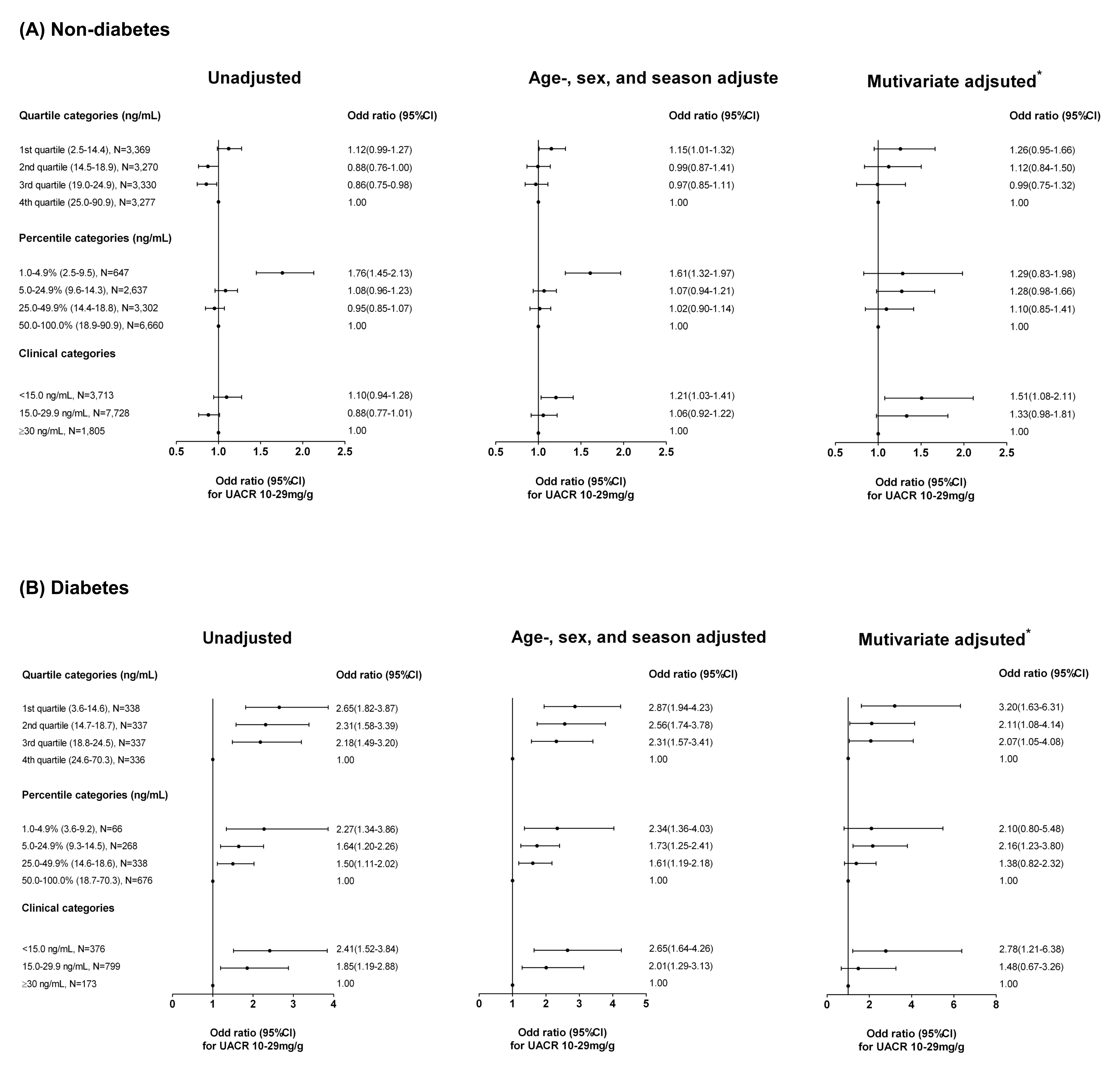

Supplement: Additional file 1: Figure S1 — Odds ratios for high-normal urinary albumin excretion (UACR 10–29 mg/g) as a function of serum 25-hydroxyvitamin D levels by quartile categories (top), percentile categories (middle), and clinical categories (bottom) in subjects without diabetes and with diabetes. Dashed lines represent 95% CIs. *Adjusted for age, sex, season, body mass index, hemoglobin, estimated glomerular filtration rate, hypertension, diabetes, use of vitamin D supplements, and/or renin-angiotensin system blocker, serum albumin, calcium, phosphorus, high-density lipoprotein-cholesterol, triglyceride, and high sensitivity-C-reactive protein. UACR, urinary albumin-creatinine ratio. [file 1471-2369-15-97-S1.tiff]
